# Knowledge of Diabetes, its Treatment and Complications Amongst Diabetic Patients in a Tertiary Care Hospital

**DOI:** 10.4103/0970-0218.42068

**Published:** 2008-07

**Authors:** Michell Gulabani, Mary John, Rajesh Isaac

**Affiliations:** Department of Community Medicine, Christian Medical College, Ludhiana, India

## Introduction

At present, India is considered as the diabetic capital of the world. There are approximately 3.5 crore diabetics in India, and this figure is expected to increase up to 5.2 crore by 2025. Every fifth patient visiting a consulting physician is a diabetic and every seventh patient visiting a family physician is a diabetic. Keeping in view the alarming increase in the incidence and prevalence of diabetics in India, the World Health Organization (WHO) has declared India as the ‘Diabetic Capital’ of the world.(1) Studies have shown that increasing patient knowledge regarding disease and its complications has significant benefits with regard to patient compliance to treatment and to decreasing complications associated with the disease.(2) Considering this, we sought to quantify in a population of diabetics visiting our clinic, the level of knowledge with respect to different areas pertaining to the prevention and treatment of associated complications.

## Materials and Methods

### Study design

We conducted a cross-sectional survey using a structured questionnaire. The subjects were diabetic patients attending the integrated diabetes clinic in Christian Medical College, Ludhiana (a university affiliated teaching hospital). The questionnaire consisted of questions that tested the patients' knowledge of diabetes, its treatment and complications. The study was conducted in July and august 2005 and was funded by Indian Council of Medical Research (ICMR).

### Sample size

Assuming that 50% of the diabetics had reasonable knowledge about various factors associated with the disease and that we require a precision of 10%, the sample size is calculated as:

$$N=4\mathrm{pq}/d^{2}=(4\times 0.5\times 0.5)/(0.1\times 0.1)=100$$

Where p is the proportion of the estimated population and q = (1-p), d representing the absolute precision.

### Data entry and analysis

We used the software Epi-lnfo Version 6.04b from CDC, Atlanta, for data entry and analysis. Percentages were calculated for descriptive statistics. Knowledge scores were calculated by cumulating points given for correct answers given by the patients. Maximum score attainable was 37. Student's *t* -test was performed to compare the mean diabetes knowledge score between relevant groups. Multiple linear regression was carried out to determine the influence of gender on the knowledge score after controlling other factors.

### Observations

There were 67 males (66.3%) and 34 females (33.7%) in the study population. In this study, 90 patients had type 2 diabetes and 11 had type 1 diabetes. Of the 101 diabetic patients, 51 (50.5%) thought that diabetes to be incurable. Forty seven (46.5%) patients thought that diabetes could be prevented. Seventy one (71.3%) patients did not know the risk factors involved in the development of diabetes. Twenty (20.7%) patients did not know their target fasting blood sugar. Thirty nine (39.6%) patients did not know their target post-prandial blood sugar.

The various responses for the questions posed to the patients regarding diabetes and its complications are given in Tables 1 and 2.

**Table 1:** Identification of organs that could be damaged by diabetes

Organ	Number of subjects identifying the organs damaged by diabetes (N = 101)
Eyes	91 (90.1%)
Heart	65 (64.4%)
Kidney	73 (72.3%)
Bones	34 (33.7%)
Feet	58 (57.4%)
Spine	8 (7.9%)

**Table 2:** Knowledge of measures that can be taken for preventing complications in diabetes

Preventive measures	Number of patients identifying the preventive measures (%) (N = 101)
Regular blood sugar testing	88 (87.1)
Regular inspection of feet	42 (41.6)
Losing excess weight	31 (30.7)

Of the 101 diabetic patients 50 (50.5%) did not know that kidney function tests should be performed in diabetes. Ninety-four (94.1%) patients did not know about glycosylated hemoglobin (HbA1c).

Of the 101 diabetic patients, 48 (48.5%) did not know about the symptoms of hypoglycemia. Yet, 77 (76.2%) knew that sweets should be consumed if they were hypoglycemic. Of the 101 patients, 11 (10.9%) said that they would definitely have taken preventive measures seriously had they known earlier that diabetes could be prevented. Sixty (59.4%) patients said that they would probably have taken preventive measures seriously.

Total knowledge scores for each patient were calculated by cumulating the scores for correct answers. The maximum score attainable was 37. The mean score in men was 2.84 points higher than that in women and the difference was found to be statistically significant (*t* -statistic = 2.44, *P* = 0.016). On applying multiple linear regression, this difference was found to be statistically significant after controlling for age, duration of disease, type of diabetic and whether the patient was private or general (*P* = 0.018). Duration of disease was also found to be significantly associated with higher knowledge score after controlling for these factors (*P* = 0.001).

## Discussion

Patients' knowledge regarding the treatment and complications of diabetes showed serious deficiencies, more so among women, even though most had been diabetic for years.

A significant predictor for lower knowledge scores was female gender. In this study, the mean score of the women was 2.84 points lower than that of men (*t* = 2.44, *P* = 0.016). This finding was similar to that reported by Vishwanathan *et al*, who conducted a study on the knowledge of diabetic subjects regarding foot problems and care of feet.(3) They demonstrated that a low knowledge score was more common among women than in men. In a study conducted in Chandigarh, it was again shown that knowledge concerning the prevention of diabetes complications was partial amongst diabetics, with only 63.3% of the diabetics taking care of their feet through regular washing.(4)

The fact that 51 (50.5%) patients thought that diabetes is curable, and that only 64 (63.4%) patients correctly said that the treatment continues throughout the life, may reflect a mentality of patients that once the blood sugars are controlled, they can stop taking their medicines. Only 47 (46.5%) correctly said that diabetes is preventable and only 29 (28.7%) were aware of the causes of diabetes. This indicates a significant lack of the knowledge of primary and primordial prevention of diabetes in the population. This fact along with that 71 of the 101 (70.3%) patients said that they would either definitely or probably have taken preventive measure seriously had they known that diabetes was preventable means that imparting knowledge regarding prevention should be a major thrust in the future.

A study from Singapore demonstrated that diabetes education had changed the practice among diabetics toward a more effective self-care.(5) The fact that although 96% of the patients were aware of how often they should have their blood sugars tested, only 60.4% were actually aware of their target fasting and post-prandial blood sugars; this also indicates an overdependence on the physician and a lack of empowerment of the patient.

Diabetes is the most common cause of non-traumatic lower limb amputations. Only 57.4% of the patients, however, knew that the feet are affected in diabetes. The most common cause of death amongst diabetics is cardiovascular disease. Only 64.4% of the subjects knew that diabetes affects the heart. Diabetes is also a leading cause of end-stage renal disease. In the present study, 26.7% of the patients did not know that diabetes affects the kidneys. The American Association of Clinical Endocrinologists states that the cause of complications in both acute and chronic diabetes is either a lack of understanding with regard to the long- and short-term regulation of blood glucose or the patient refusing to control the blood glucose levels.(6)

One of the most optimum tests for evaluating long-term blood sugar control is HbA1c. Although this test is conducted in the hospital for these patients, only 5.9% actually knew that such a test is available. Studies have shown that patients with a better knowledge of HbA1c were able to more accurately regulate their diabetic status.(2)

In our study, only 52 (51.5%) patients actually knew the symptoms of hypoglycemia. However, 77 (76.2%) patients knew that they should consume sweets if they had experienced an episode of hypoglycemia. This shows that the knowledge of diabetes in patients is only partial and that most patients may not be able to take appropriate corrective measures sufficiently early and may seek medical aid only at very late stages.

In this study, the role of gender on knowledge regarding diabetes was evident with women scoring significantly lower than men even after regulating other confounders. This implies that an extremely targeted education program is required to empower diabetic women.

This study confirms that patient knowledge about the treatment and complications of diabetes is limited, especially with regard to preventive aspects. There is a definite need to empower patients with the knowledge required to help them obtain maximum benefit from their treatment for diabetes.
